# Patient Perspectives on IUD Placement: The Impact of a MyChart Message

**DOI:** 10.1007/s11606-025-09907-7

**Published:** 2025-10-31

**Authors:** Alekses Clifton, Danielle Cook, Morgan M. Millar, Vidya Gopinath

**Affiliations:** 1 Department of Internal Medicine, Division of General Internal Medicine, University of Utah, Salt Lake City, UT USA; 2 Department of Internal Medicine, Division of Epidemiology, University of Utah, Salt Lake City, UT USA

## INTRODUCTION

Intrauterine device (IUD) placement can be painful, and fear of pain may lead patients to avoid this form of contraception.^1^ Besides pre-procedural anxiety, many women report their procedural pain was dismissed by clinicians. National media coverage has highlighted instances of inadequate analgesia for women undergoing these procedures.^2,3^ Current discourse in medical literature and popular media highlights the need to make the IUD insertion process less traumatizing and more patient-centered. The CDC and the American College of Obstetrics and Gynecology (ACOG) recently updated recommendations, highlighting the need for improved processes and pain management.^4–6^ Studies for other outpatient procedures and small studies on IUD placement have indicated a significant benefit with appropriate guidance and counseling.^7^ In this study, we aimed to measure peri-procedural pain and anxiety in women presenting for IUD insertion after implementing a patient-centered protocol with an emphasis on anticipatory guidance.

## METHODS

We performed an observational cohort survey study in women undergoing IUD insertion at two Internal Medicine clinics between December 2024 and June 2025 to evaluate experiences with our new patient-centered protocol. The protocol included a pre-procedure MyChart or phone message, NSAIDs, optional benzodiazepine prescription, and administration of a cervical lidocaine block. Patients scheduled for IUD placement received a MyChart message within 72 h of their appointment. The standardized message recommended taking ibuprofen 800 mg approximately 30 to 60 min prior to the appointment, offered 0.5 mg lorazepam, and outlined the visit including an opportunity to ask questions, a formal consent process, and a urine pregnancy test.

Patients requesting a benzodiazepine were scheduled for a virtual visit with the performing physician within 48 h prior to answer questions, obtain consent, and explain logistics (e.g., need for a driver). Immediately after the procedure, patients completed a post-procedure survey with approximately 15 questions related to anticipatory guidance, anxiety, and pain (before, during, and after the procedure).

### Statistical Analysis

Demographics, pain, and anxiety results were summarized for the cohort using frequency and percent for categorical variables and mean and standard deviation (SD) or median and interquartile range (IQR) for continuous variables.

## RESULTS

### Patient Characteristics

A total of 29 participants underwent IUD insertion and completed the study survey (median age was 29 years [IQR 22, 40]). Most patients opted for NSAIDs and the lidocaine block. Only 17% accepted a benzodiazepine. Twenty-one participants (72%) opted for multiple treatment options (Table 1).

**Table 1:** Patient Characteristics, Pain Relief Options, and Outcomes (*N* = 29)

Characteristic	Freq. (%)
Age (*median (IQR)*)	29 (22–40)
Optional medications*	
NSAIDs	24 (83%)
Lidocaine block	26 (90%)
Benzodiazepine	5 (17%)
Prior IUD	14 (48%)
Anxiety prior to procedure	
Not at all anxious	2 (7%)
A little anxious	6 (21%)
Moderately anxious	13 (45%)
Very anxious	7 (24%)
Extremely anxious	1 (3%)
Expected pain during procedure (*mean (SD)*)	6.2 (2.55)
Pain during procedure (*mean (SD)*)	6.2 (2.01)
Pain immediately following procedure (*mean (SD)*)	3.1 (2.22)

*Participants could choose to receive more than one medication

### Procedure-Related Pain and Anxiety

The mean level of anticipated pain was 6.2 out of 10 (SD 2.6), the same as the mean actual pain experienced during the procedure (mean, 6.2; SD, 2.0; Table 1). Prior to the procedure, 72% were moderately to extremely anxious. Of the five participants receiving a benzodiazepine, three were moderately anxious and two were very anxious.

### Patient-Centered Protocol

**Figure 1:**
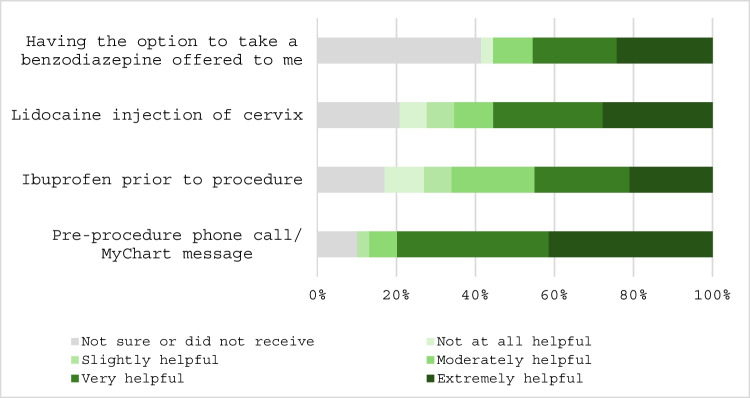
How helpful participants rated each aspect of the protocol

Participants were asked to report the helpfulness of protocol elements leading up to their IUD procedure. The most helpful element was the pre-procedure MyChart message/phone call (79% very or extremely helpful) (Fig. 1). Nearly half (45%, 13/29) of participants indicated the option to take a benzodiazepine was very to extremely helpful, although few accepted it.

## DISCUSSION

This study evaluated patient-reported pain and perceptions of “helpfulness” of supportive interventions for IUD placement. Most notably, patients identified the anticipatory guidance message as the most helpful intervention. While the offer of a benzodiazepine was rated highly by almost half of our participants, only five patients accepted it.

Recent efforts to improve the patient experience around IUD placement have often focused on reducing pain. Utilizing our patient-centered protocol, the participants were able to accurately gauge how much pain they could expect during the procedure. It also prioritized patient-reported perceptions of “helpfulness,” providing a broader understanding of what interventions patients value. Although real-time counseling during procedures is widely acknowledged as important, this highlights the added value of pre-procedure counseling and anticipatory guidance.

A strength of this study is its integration into routine care in clinics, reflecting patient-centered primary care. However, limitations include the small sample size, absence of a control group, and the involvement of only two physicians, which may limit generalizability. Future studies should further expand the effectiveness and perceived value of patient-centered interventions to improve procedural experiences in primary care.
